# Supplementary material for: Extracellular nanovesicles for packaging of CRISPR-Cas9 protein and sgRNA to induce therapeutic exon skipping
Source: Nat Commun. 2020 Mar 13;11:1334. doi: 10.1038/s41467-020-14957-y (PMC7070030; doi:10.1038/s41467-020-14957-y)
Supplement: Supplementary file 1 — Supplementary Information [file 41467_2020_14957_MOESM1_ESM.pdf]

Supplementary Figure 1.

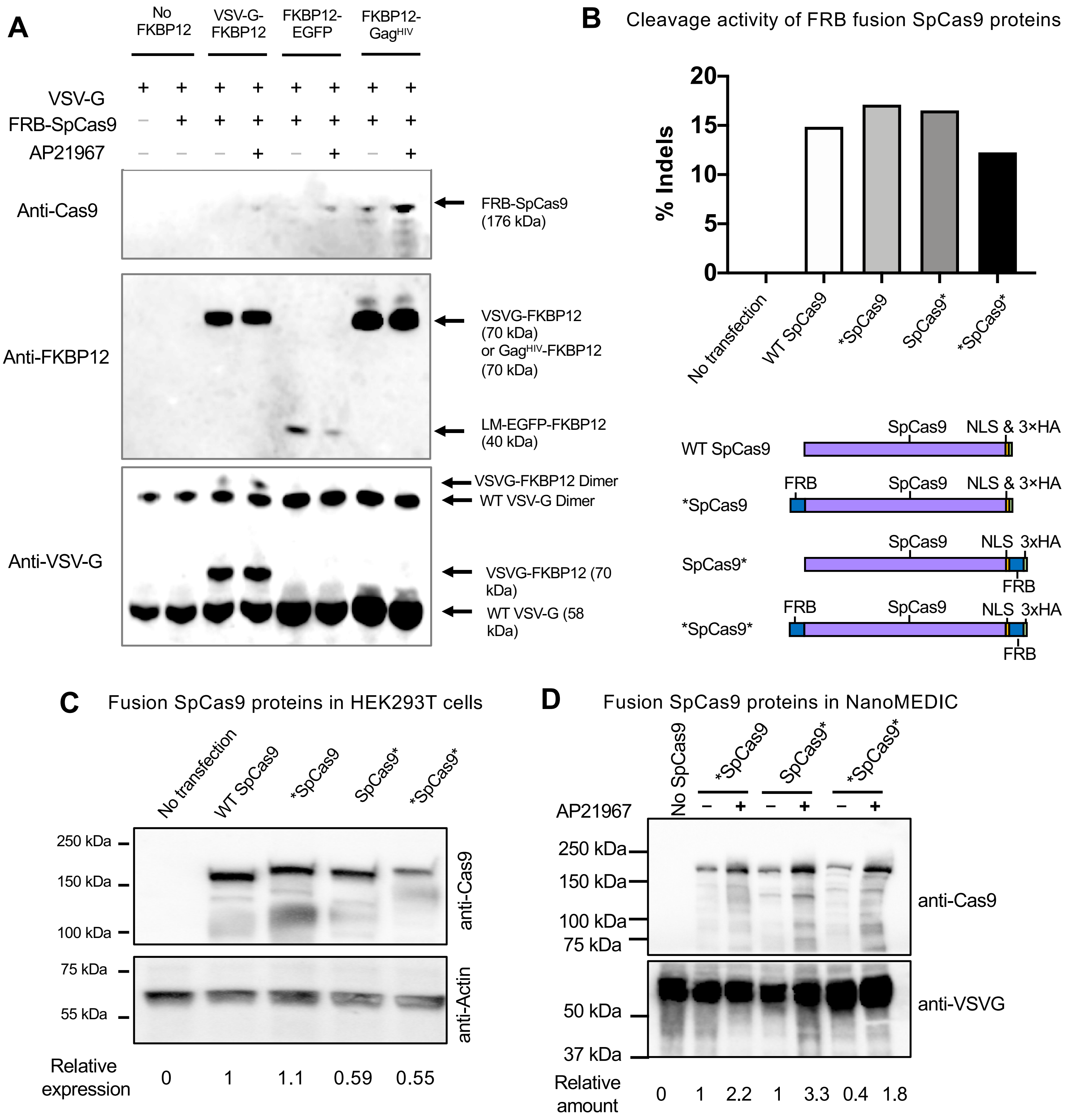

Supplementary Figure 1. Incorporation of FRB-fused SpCas9 protein into NanoMEDIC.
(**A**) Western blot analysis of EVs containing FRB-SpCas9 and no FKBP12 interaction partner, VSV-G-FKBP12, FKBP12-EGFP-A, or FKBP12-Gag^{HIV} in the presence or absence of AP21967, used in Figure 1. VSV-G protein amount was also examined as a loading control of EVs. Results are representative of two independent western blot images from two independent experiments. (**B**) Cleavage activity of FRB-SpCas9 fusion constructs. The mutation frequency of WT SpCas9, FRB N-terminal SpCas9 (*SpCas9), FRB C-terminal SpCas9 (SpCas9*), and FRB N- and C- terminal SpCas9 (*SpCas9*) activity were assessed in HEK293T cells transfected with the fusion constructs and DMD1-sgRNA. gDNA was extracted 3 days after transfection and analyzed by T7E1 assay. (C) Relative expression of SpCas9 protein 3 days post transfection was determined by western blot quantification normalized by actin (ACTB) protein. Results are representative of two independent western blot images from a single experiment. (**D**) Western blot analysis of NanoMEDIC generated in the presence or absence of AP21967 with *SpCas9, SpCas9*, or *SpCas9*. Relative amount SpCas9 was determined by dividing the density of the SpCas9 band by that of the VSV-G band. Results are representative of two independent western blot images from a single experiment.

Supplementary Figure 2.

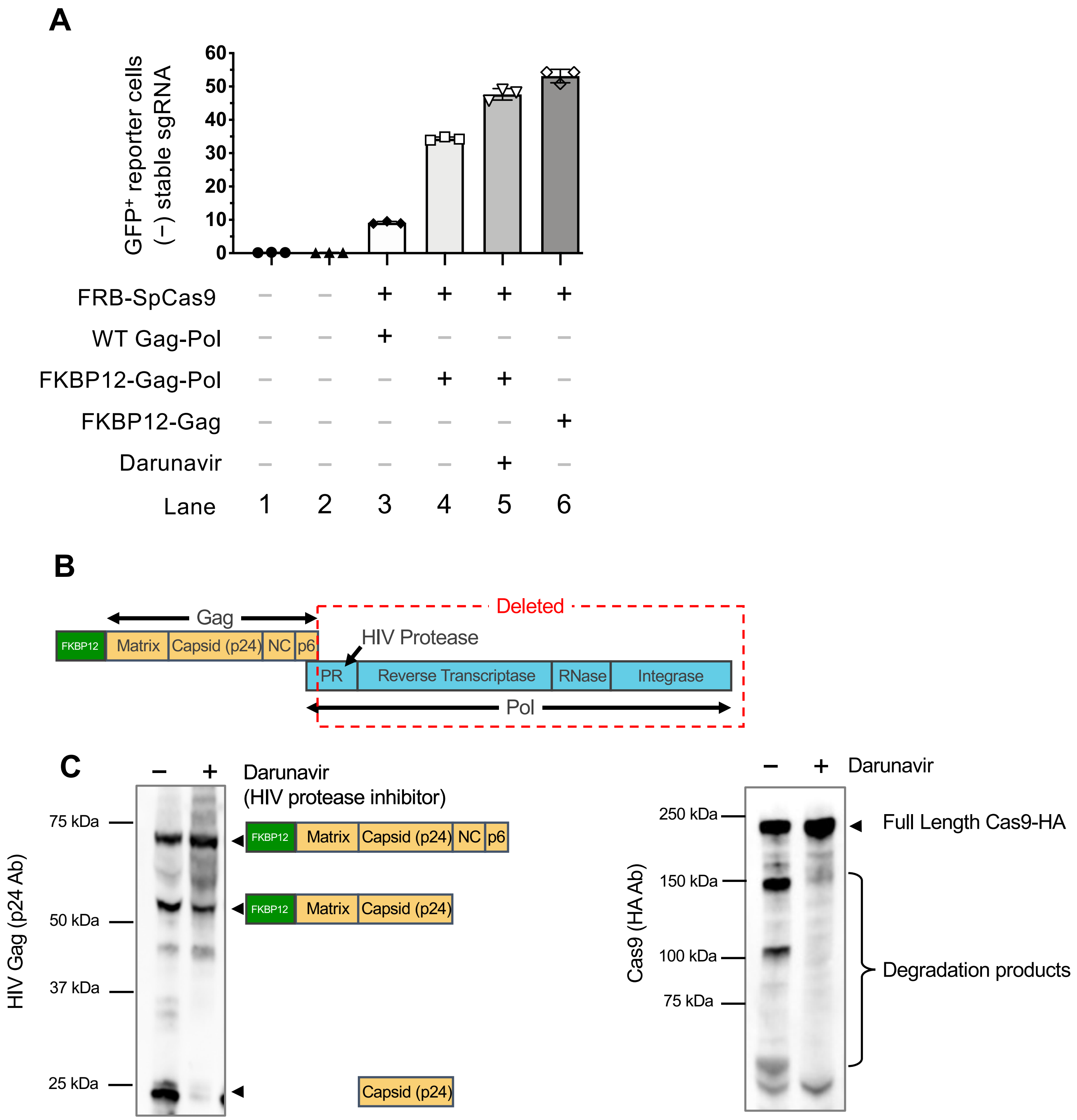

Supplementary Figure 2. NanoMEDIC is more efficient at delivering SpCas9 RNP complexes compared with other dimerization SpCas9 packaging system.

(**A**) HIV protease reduces delivery of active RNP complexes to recipient cells. All-in-one NanoMEDIC containing different anchoring Gag constructs were generated in the following conditions: Lane 1, no EVs; Lane 2, VSV-G and FRB-SpCas9; Lane 3, WT Gag-pol^{HIV} and FRB-SpCas9; Lane 4, FKBP12-Gag-pol^{HIV} and FRB-SpCas9 without Darunavir; Lane 5, FKBP12-Gag-pol^{HIV} and FRB-SpCas9 with Darunavir; Lane 6, FKBP12- Gag^{HIV} and FRB-SpCas9 without Darunavir. The resulting NanoMEDIC particles were inoculated onto HEK293T EGxxFP reporter cells and analyzed by flow cytometry 3 days after inoculation. Mean ± S.D. from technical triplicates.

(**B**) Schematic depicting the Gag-Pol and the deleted portion to yield FKBP12-Gag^{HIV}.

(**C**) Western blot analysis of NanoMEDIC generated with FKBP12-Gag-pol^{HIV} in the presence or absence of the HIV-1 protease inhibitor, Darunavir. Antibodies against Gag-pol^{HIV} and SpCas9 were used. Degradation products of SpCas9 without Darunavir indicates HIV protease non-specifically cleaves SpCas9. Results are representative of two western blots from two independent NanoMEDIC preps.

Supplementary Figure 3.

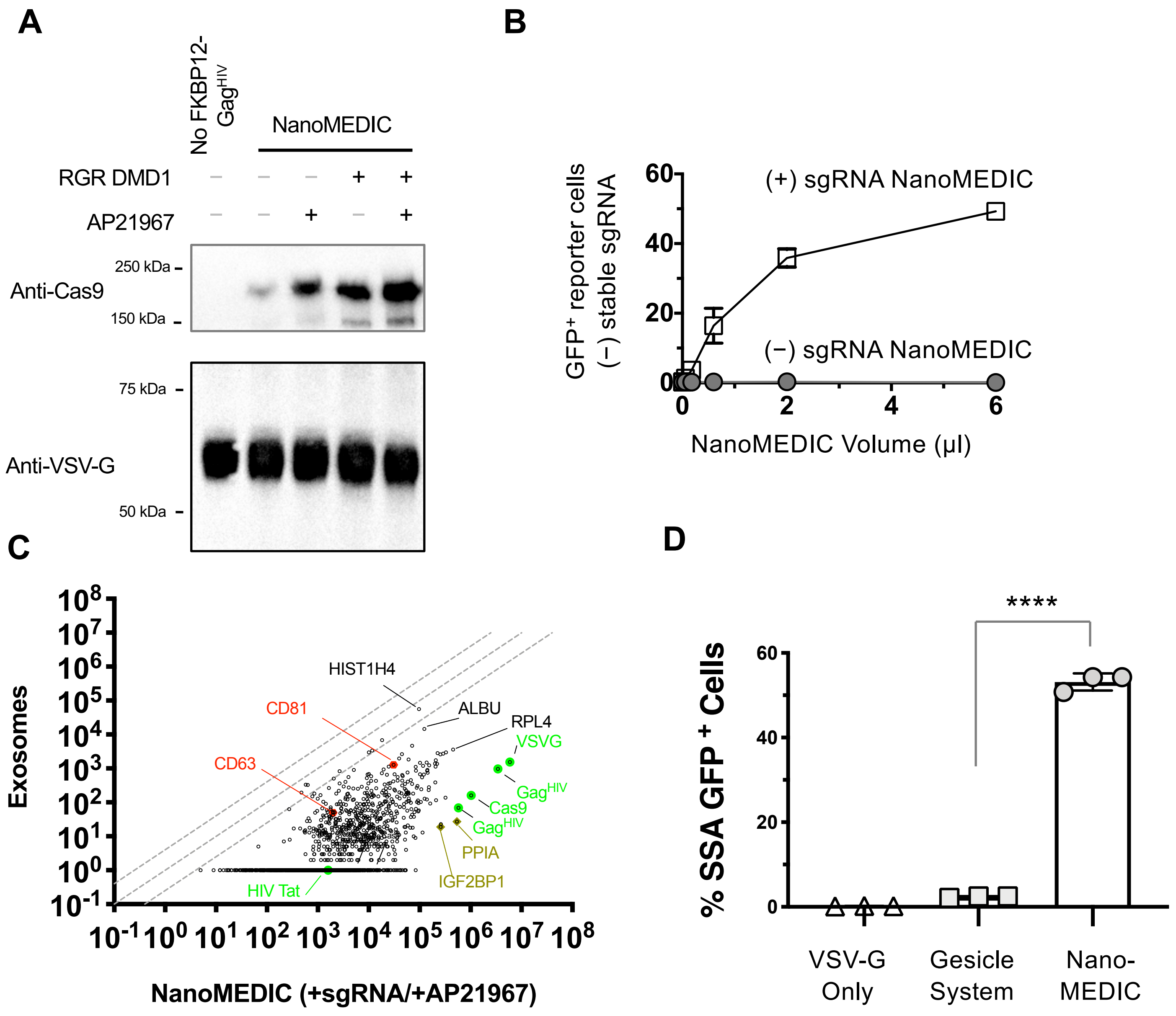

Supplementary Figure 3. Proteomic characterization of NanoMEDIC particles.
(**A**) AP21967 and sgRNA expression synergistically recruit FRB-SpCas9 into NanoMEDIC. Western blot of FRB-SpCas9 protein contained in the NanoMEDIC particles. Western results are representative of two western blots from two independent NanoMEDIC preps.
(**B**) All-in-one concentrated NanoMEDIC particles containing RGR-DMD1 or no sgRNA were inoculated onto HEK293T EGxxFP reporter cells at the indicated volumes. The results show SSA-GFP+ expression measured by flow cytometry analysis 3 days after inoculation. Mean ± S.D. from technical triplicates.
(**C**) A comparison of proteins contained in exosomes released from HEK293T cells compared with NanoMEDIC produced with AP21967 and RGR-DMD1. The scatter plot shows NanoMEDIC proteins in green (derived from components of the NanoMEDIC VSV-G envelope, SpCas9 protein, and Gag^{HIV}, and Tat), common exosome related proteins in red (CD63 and CD81), and other enriched proteins associated with Gag in yellow (IGF2BP1 and PPIA).
(**D**) Commercially available Guide-It Clontech gesicles and NanoMEDIC were produced side-by-side at the same scale and inoculated onto HEK293T EGxxFP reporter cells. Three days after inoculation, cleavage activity was analyzed for SSA-GFP+ expression by flow cytometry. Flow cytometry results of HEK293T EGxxFP cells inoculated with VSV-G only EVs, Guide-It Clontech gesicles, or NanoMEDIC are depicted in the bar graph. ****, $P < 0.0001$ by unpaired two-tailed *t*-test. Mean ± S.D. from technical triplicates.

Supplementary Figure 4.

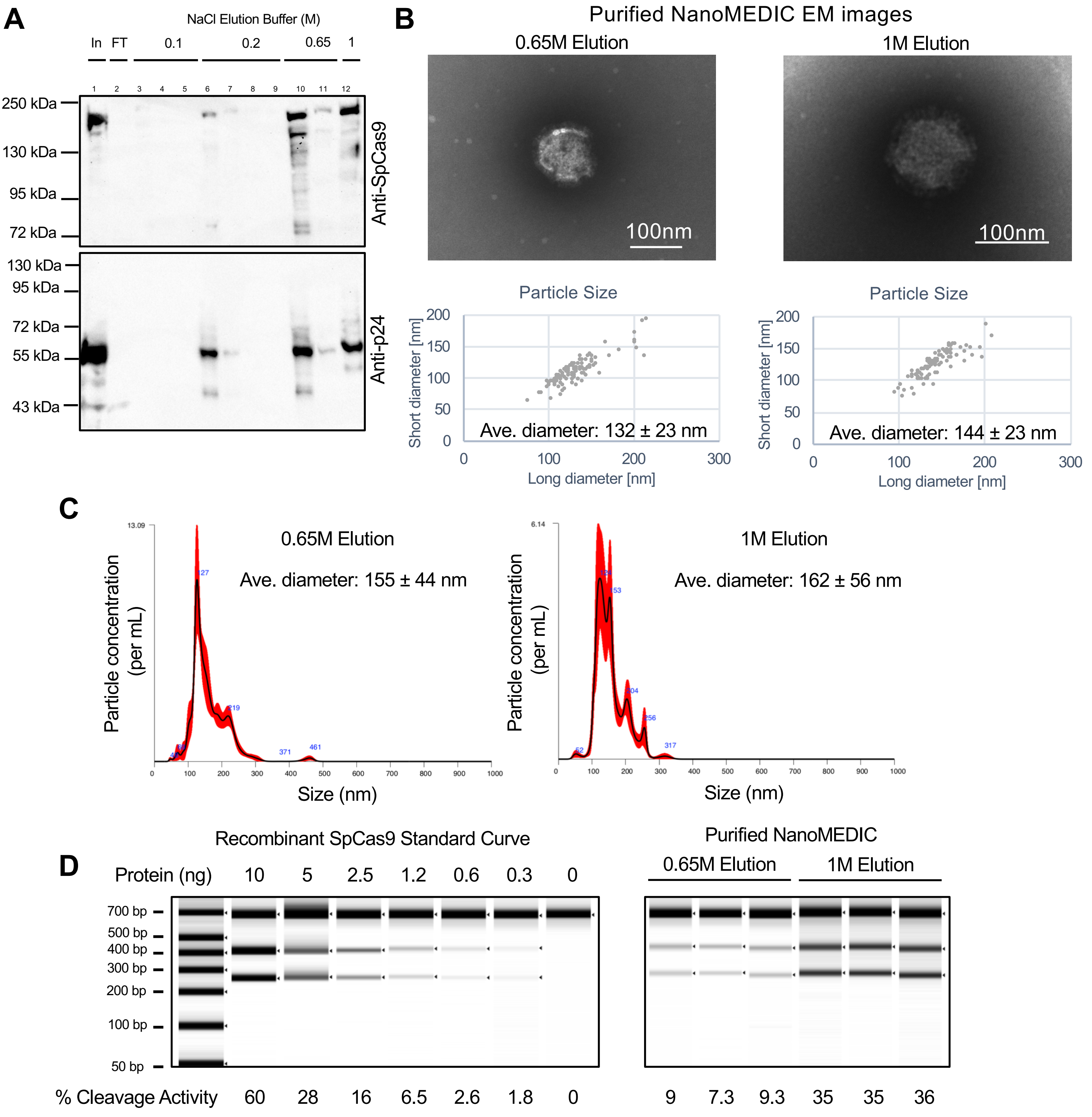

E

Elution condition	EM diameter	NanoSight diameter	NanoSight particle No. per mL	Active Cas9 protein	Active Cas9 molecules per mL	Active Cas9 molecules per particle
0.65 M	132 ± 23 nm	155 ± 44 nm	3.00E+12	3.4 μg/ml	1.20E+13	3.5
1.0 M	144 ± 23 nm	162 ± 56 nm	6.00E+12	13.3 μg/ml	4.80E+13	7.9

Supplementary Figure 4. Characterization of NanoMEDIC particles.

(**A**) NanoMEDIC targeting DMD1 was purified by affinity chromatography and eluted by increasing amounts of NaCl. Western blot analysis was performed elution samples for SpCas9 and GagHIV to determine which fractions NanoMEDIC was eluted in. Two elutions were collected at 0.65 M and 1 M NaCl concentrations.

(**B**) 0.65 M and 1 M elution samples were analyzed by electron microscopy and the particle size distribution was quantified by image J analysis. Results are representative of a single electron microscopy experiment analyzing a single prep of purified NanoMEDIC particles. 109 particles from 0.65 M NaCl elution purified NanoMEDIC were analysed while 90 particles from 1 M NaCl elution purified NanoMEDIC were analysed.

(**C**) 0.65 M and 1 M elution samples were analyzed by NanoSight tracking analysis to determine the size distribution of purified NanoMEDIC particles. Western results are representative of three independent purification preps of NanoMEDIC.

(**D**) The amount of active RNP complexes contained in concentrated 0.65 M and 1 M elution samples were analyzed an *in vitro* cleavage assay using a PCR amplified DNA template containing the sgRNA targeting site. A recombinant SpCas9 standard curve with *in vitro* transcribed sgRNA was used to calculate the amount of active RNP complexes contained in NanoMEDIC particles. Results are representative of a single experiment performed in technical triplicate from a large 600 mL purification of NanoMEDIC.

(**E**) Summary of the nanoparticle analyses and estimation of active Cas9/sgRNA complex number per particle.

Supplementary Figure 5. NanoMEDIC targets various cell types.

(**A**) Jurkat cells were inoculated with NanoMEDIC targeting the CCR5 locus. T7E1 analysis of indels induced at the CCR5 locus is depicted. Results in Jurkat cells inoculated with NanoMEDIC was performed in technical triplicates. Similar results were obtained in an independent experiment in HEK293T cells inoculated with the same prep of NanoMEDIC targeting the CCR5 locus.

(**B**) U937 monocytes stably expressing EGFP were inoculated with NanoMEDIC targeting EGFP. As an EGFP negative control, U937 cells stably expressing SAMHD1 were used. Flow cytometry analysis was performed 3 days after inoculation, and EGFP MFI (mean fluorescence intensity) was reduced nearly 50% compared with non-treated control. Mean ± S.D. from technical triplicates.

(**C**) iPSCs were differentiated into cortical neurons by overexpression of neurogenin2 (NGN2 IRES mCherry) from Dox-inducible *piggyBac* vector, and then inoculated with NanoMEDIC targeting two SAMHD1 sites. Genomic cleavage activity was measured by T7E1 analysis of cortical neurons treated with NanoMEDIC targeting two SAMHD1 sites in exon 1. Fluorescence microscope image is representative 14 images of cortical neurons differentiated from iPSCs in 3 independent experiments.

(**D**) T7E1 analysis of HEK293T cells transfected with SpCas9 and sgRNA expression plasmids or inoculated with 50, 200, or 500 ng of NanoMEDIC RNPs targeting DMD1, DMD23, EMX1, SAMHD1#1, SAMHD1#2, or CCR5. Mean ± S.D. from technical triplicates.

Supplementary Figure 5.

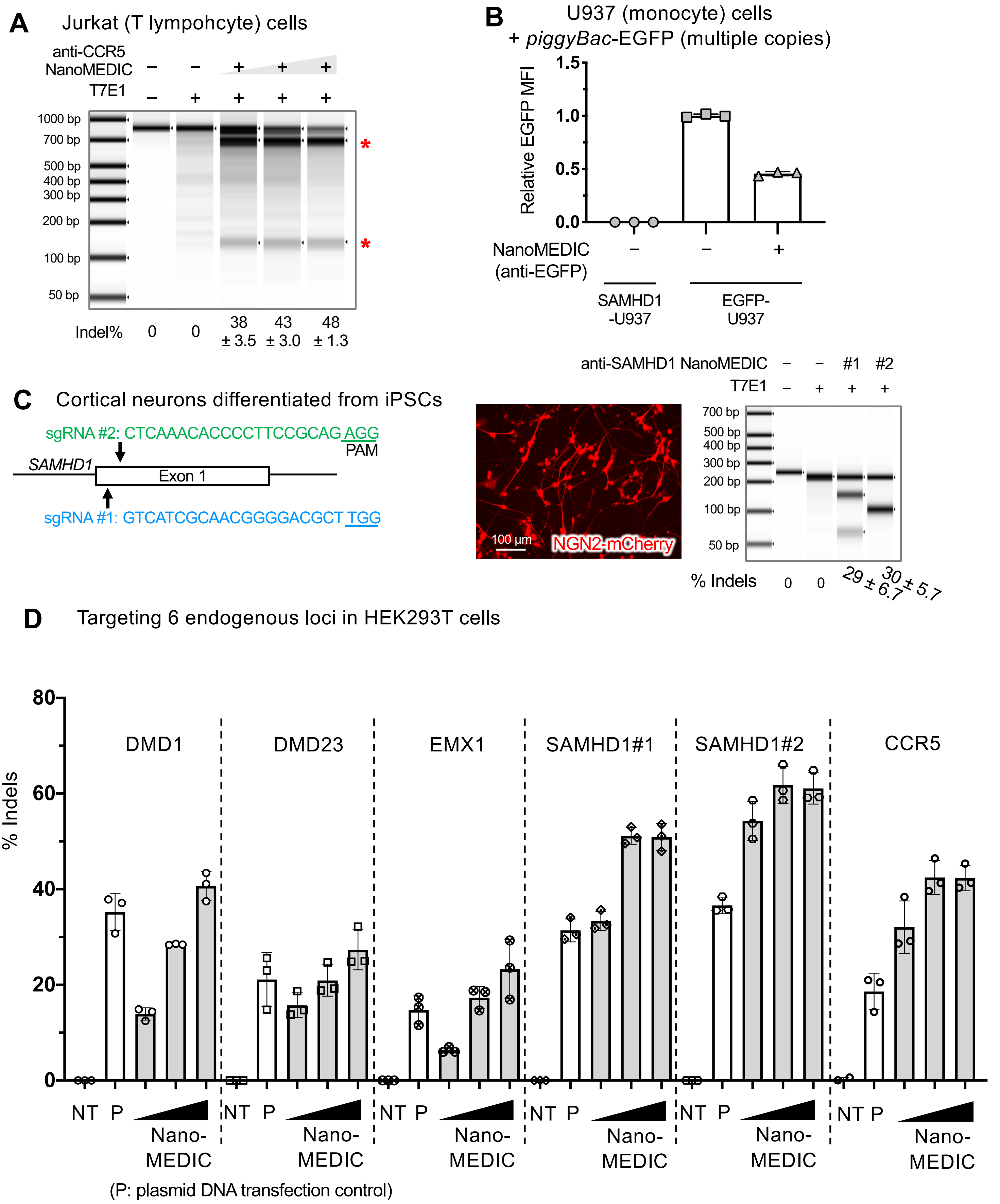

Supplementary Figure 6.

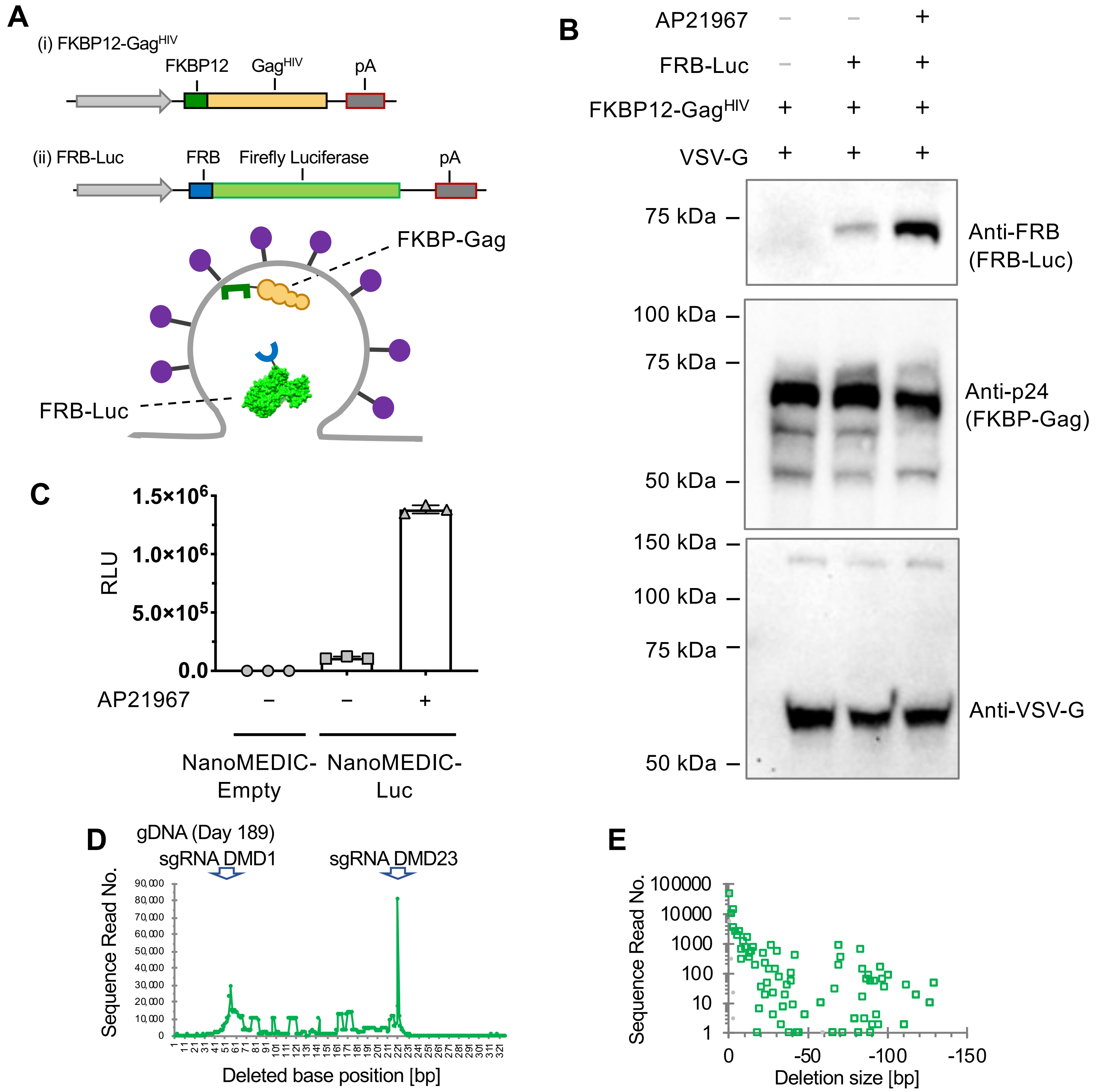

Supplementary Figure 6. NanoMEDIC for luciferase protein delivery and in vivo cleavage analysis.
(**A**) Schematic representation of DNA constructs expressing luciferase protein fused with FRB (FRB-Luc) and HIV Gag protein fused with FKBP12, as well as incorporation of both components into a budding NanoMEDIC particle.
(**B**) Western blot analysis of luciferase protein from NanoMEDIC produced indicated conditions. Western results are from a single western blot experiment from one prep of NanoMEDIC containing luciferase protein.
(**C**) HEK293T cells seeded in a 96-well plate were inoculated with NanoMEDIC produced in the indicated conditions. Cells were lysed and analyzed for luciferase activity 16 hours after inoculation. Mean ± S.D. from technical triplicates.
(**D**) Deep sequencing analysis of the genomic DNA from the Luc exon skipping reporter mice on 189 days post-injection of NanoMEDIC-Cas9 revealed sharp deletion peaks at the target sites of sgRNA DMD1 and DMD23, respectively.
(**E**) Deletion size analysis from the deep sequencing of the genomic DNA of the Luc reporter mice confirmed abundance of small (< 50 bp) deletions, as well as large (>100 bp) deletion corresponding the two cuts by sgRNA DMD1 and DMD23.

Supplementary Figure 7.

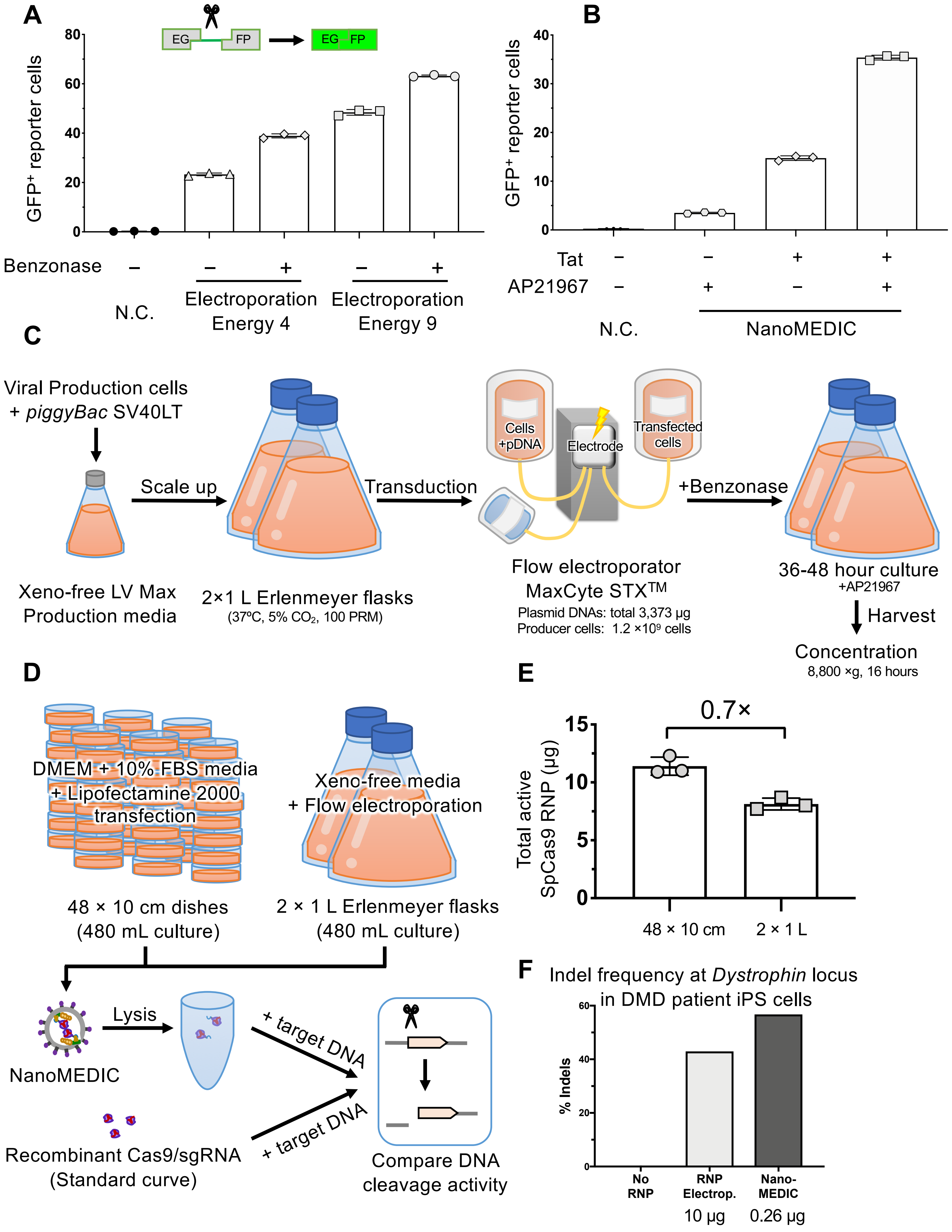

Supplementary Figure 7. Development of a suspension-based xeno-free NanoMEDIC production system by flow electroporation.

(**A**) NanoMEDIC produced by two different electroporation energies, E4 and E9, in the presence or absence of benzonase were inoculated onto HEK293T EGxxFP reporter cells and then analyzed for SSA-GFP+ expression by flow cytometry 3 days after inoculation. Mean ± S.D. from technical triplicates.

(**B**) NanoMEDIC produced by electroporation with indicated conditions were inoculated onto HEK293T EGxxFP reporter cells and then analyzed for SSA-GFP+ expression by flow cytometry 3 days after inoculation. Mean ± S.D. from technical triplicates.

(**C**) Schematic demonstrating the flow electroporation of suspension HEK293T cells by a MaxCyte STX electroporation instrument and collection of NanoMEDIC 36-48 hours later.

(**D**) Schematic showing NanoMEDIC produced by adherent culture versus suspension culture.

(**E**) Quantification of the total amount of active RNP complex in NanoMEDIC produced by adherent culture with lipofectamine transfection or suspension culture based MaxCyte electroporation, measured by an *in vitro* cleavage assay based on a recombinant SpCas9 standard curve. Mean ± S.D. from technical triplicates.

(**F**) Indel analysis of DMD patient iPSCs electroporated with 10 μg RNP by 4D-nucleofector[19] or inoculated with NanoMEDIC containing 0.26 μg active RNP complex. The sgRNA targets the DMD1 site.

Supplementary Figure 8. Uncropped original western blot images.

Figure 4e

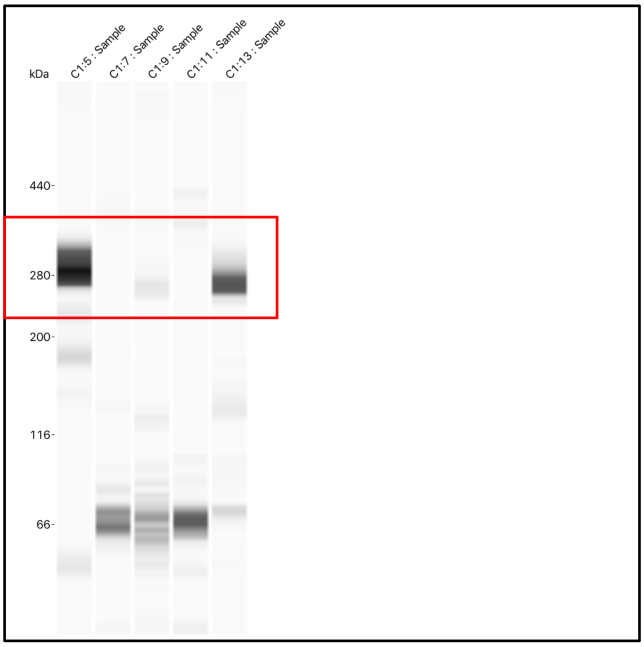

Protein Wes IB: Dystrophin

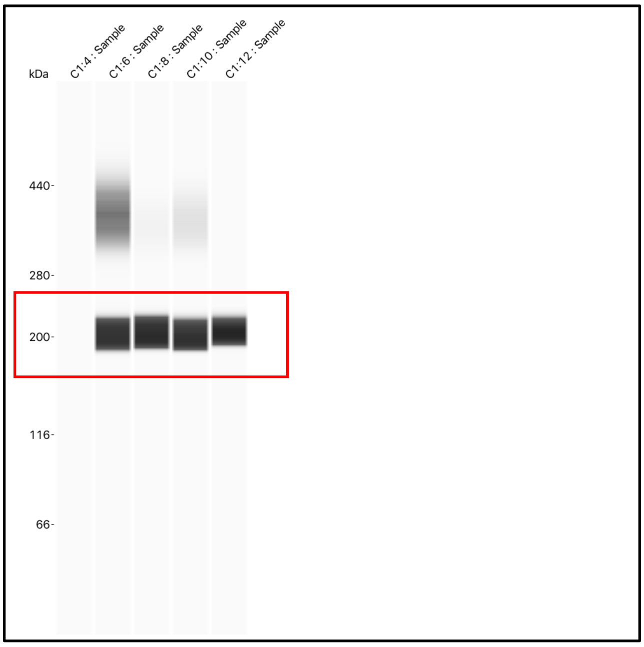

Protein Wes IB: Myosin

Figure S1A

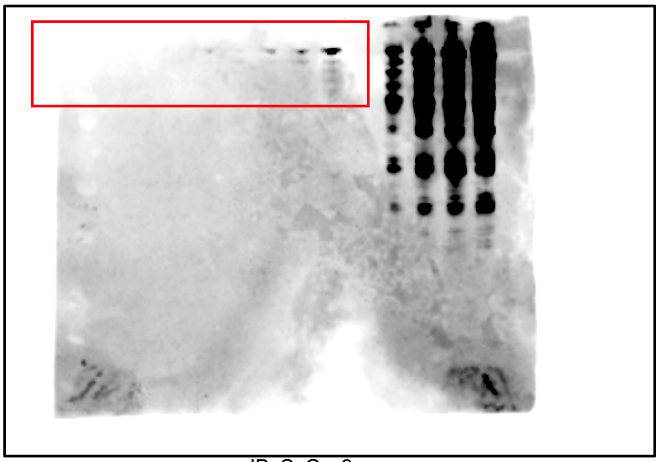

IB: SpCas9

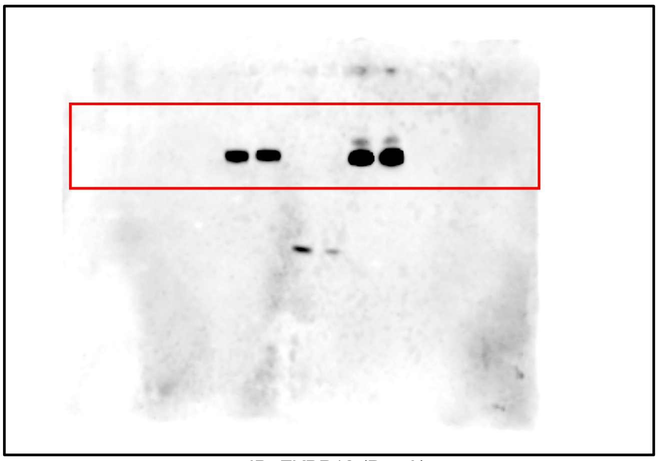

IB: FKBP12 (DmrA)

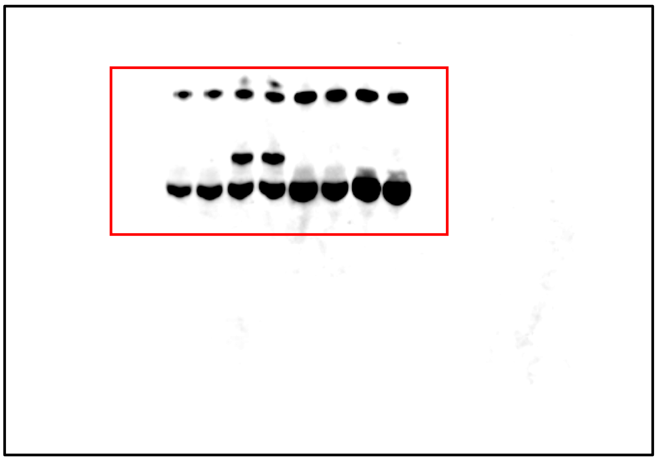

IB: VSV-G

Supplementary Figure 8. Uncropped original western blot images.

Figure S1C

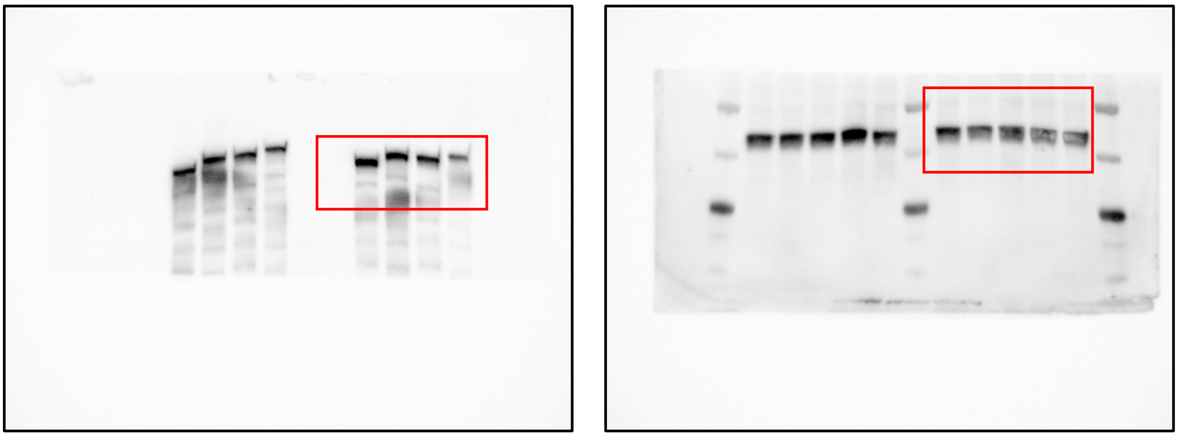

IB: SpCas9 IB: Actin

Figure S1D

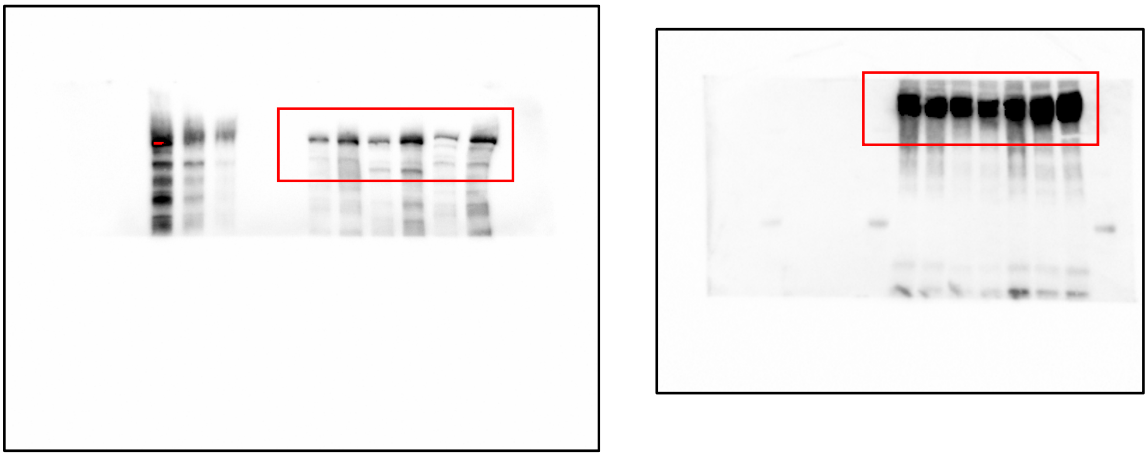

IB: SpCas9 IB: VSV-G

Supplementary Figure 8. Uncropped original western blot images.

Figure S2D

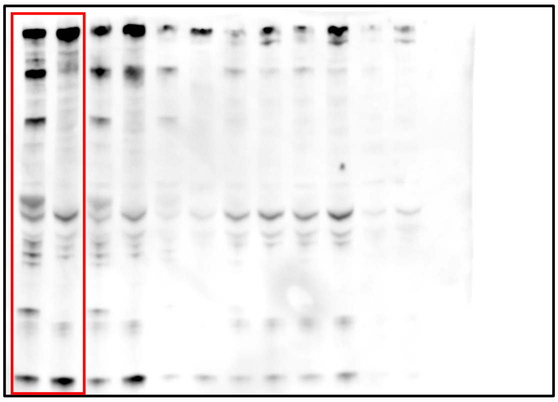

IB: HA

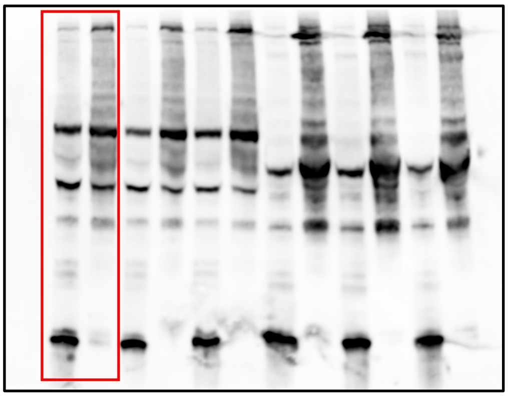

IB: HIV p24

Figure S3A

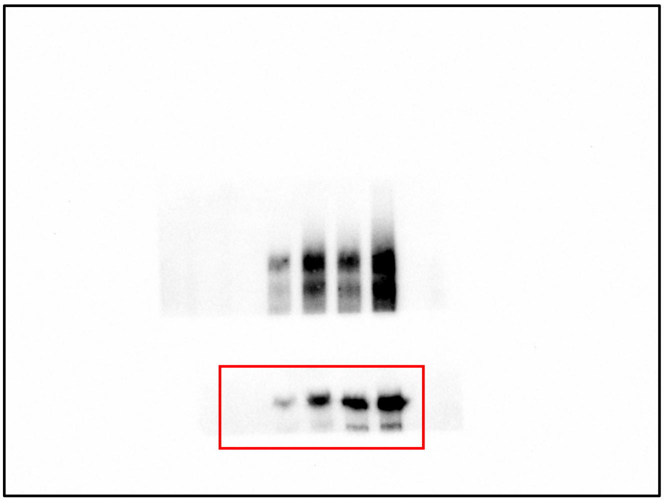

IB: SpCas9

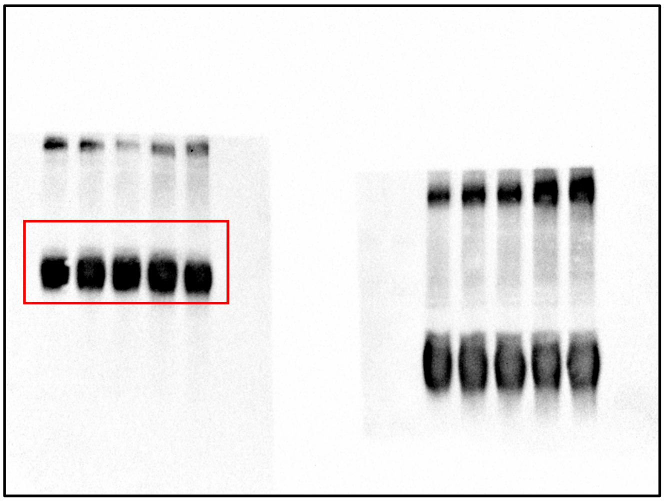

IB: VSV-G

Figure S4A

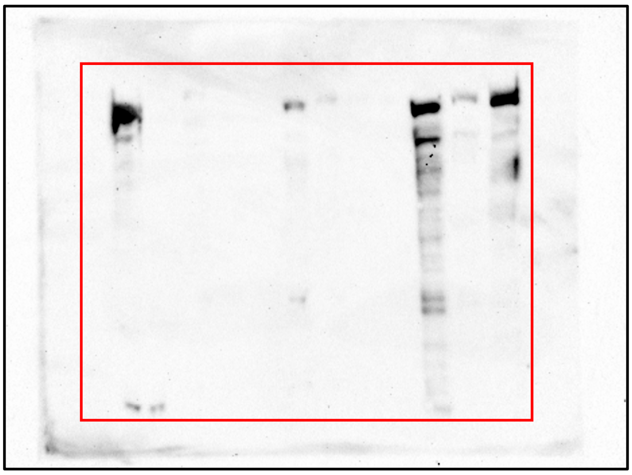

IB: SpCas9

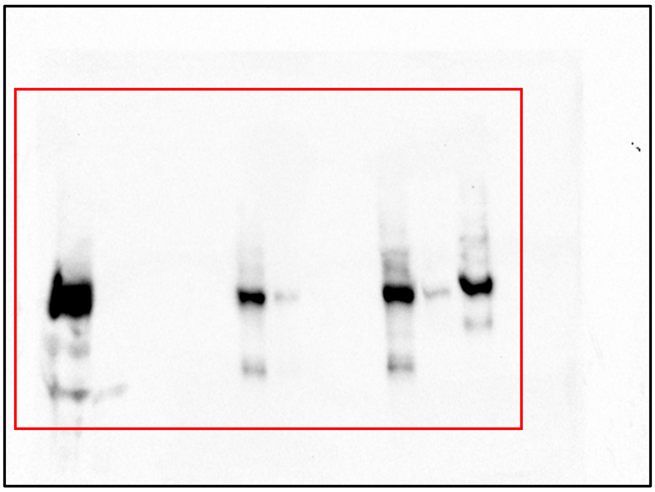

IB: HIV p-24

Supplementary Figure 8. Uncropped original western blot images.

Figure S6B

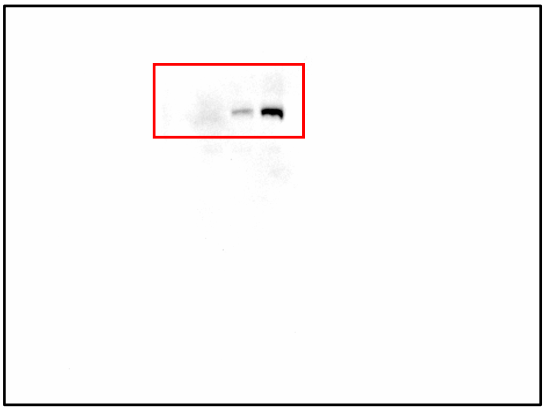

IB: FRB (DmrC)

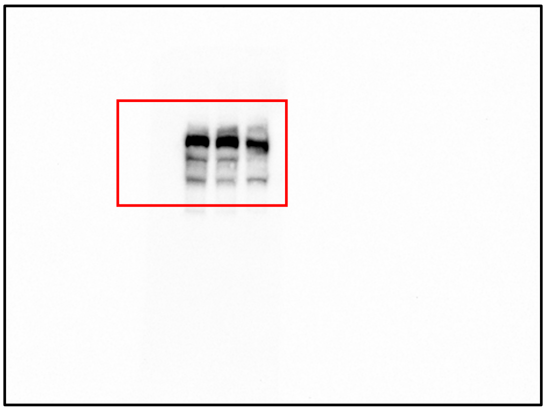

IB: HIV p24

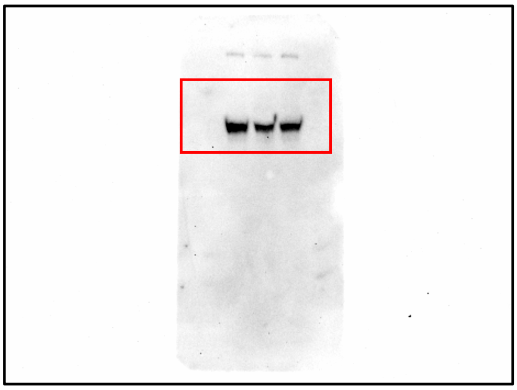

IB: VSV-G

Supplementary Figure 9.

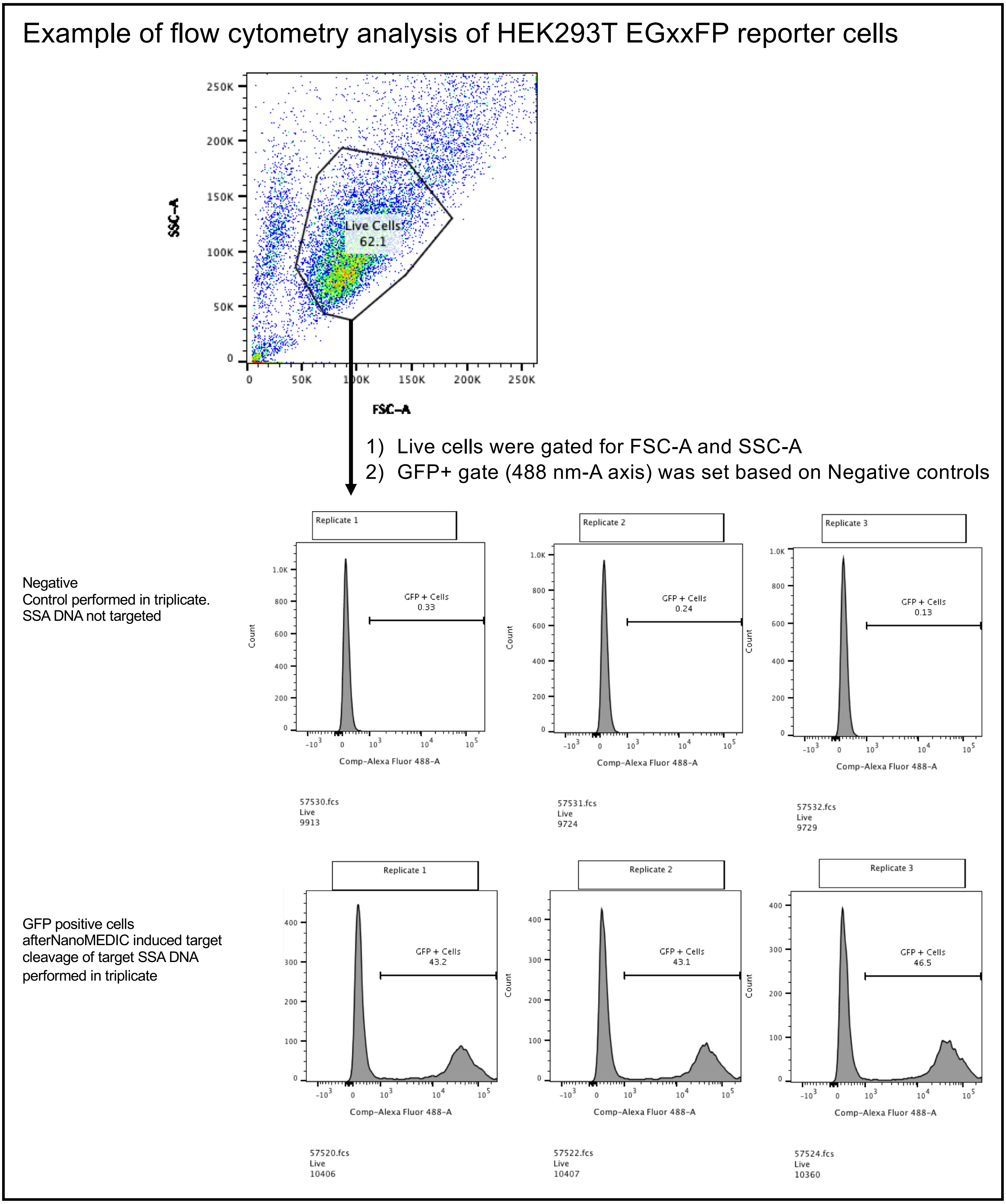

Supplementary Figure 9. Gating strategy of flow cytometry data for EGxxFP SSA reporter cells. Example of flow cytometry analysis for HEK293T EGxxFP reporter cells. First, live cells were gated for FSC-A and SSC-A to remove cell debris. Then, GRP+ gate was set based on negative controls.
