# Supplementary material for: Extracellular nanovesicles for packaging of CRISPR-Cas9 protein and sgRNA to induce therapeutic exon skipping
Source: Nat Commun. 2020 Mar 13;11:1334. doi: 10.1038/s41467-020-14957-y (PMC7070030; doi:10.1038/s41467-020-14957-y)
Supplement: Supplementary file 2 — Reporting Summary [file 41467_2020_14957_MOESM2_ESM.pdf]

nature research

Corresponding author(s): Akitsu Hotta

Last updated by author(s): 2/3/2020

Reporting Summary

Nature Research wishes to improve the reproducibility of the work that we publish. This form provides structure for consistency and transparency in reporting. For further information on Nature Research policies, see Authors & Referees and the Editorial Policy Checklist .

Statistics

For all statistical analyses, confirm that the following items are present in the figure legend, table legend, main text, or Methods section.

n/a	Confirmed	
☐	☒	The exact sample size (n) for each experimental group/condition, given as a discrete number and unit of measurement
☐	☒	A statement on whether measurements were taken from distinct samples or whether the same sample was measured repeatedly
☐	☒	The statistical test(s) used AND whether they are one- or two-sided<br>*Only common tests should be described solely by name; describe more complex techniques in the Methods section.*
☒	☐	A description of all covariates tested
☒	☐	A description of any assumptions or corrections, such as tests of normality and adjustment for multiple comparisons
☒	☐	A full description of the statistical parameters including central tendency (e.g. means) or other basic estimates (e.g. regression coefficient) AND variation (e.g. standard deviation) or associated estimates of uncertainty (e.g. confidence intervals)
☒	☐	For null hypothesis testing, the test statistic (e.g. F, t, r) with confidence intervals, effect sizes, degrees of freedom and P value noted<br>*Give P values as exact values whenever suitable.*
☒	☐	For Bayesian analysis, information on the choice of priors and Markov chain Monte Carlo settings
☒	☐	For hierarchical and complex designs, identification of the appropriate level for tests and full reporting of outcomes
☒	☐	Estimates of effect sizes (e.g. Cohen's d, Pearson's r), indicating how they were calculated

Our web collection on statistics for biologists contains articles on many of the points above.

Software and code

Policy information about availability of computer code

Data collection	Not applicable in the current version of the manuscript
Data analysis	GraphPad Prism 7 was used for data analysis. ImageJ 1.52o was used for EM analysis. ProteinPilot v5.0 and Mascot v2.5 were used for proteomic data.

For manuscripts utilizing custom algorithms or software that are central to the research but not yet described in published literature, software must be made available to editors/reviewers. We strongly encourage code deposition in a community repository (e.g. GitHub). See the Nature Research guidelines for submitting code & software for further information.

Data

Policy information about availability of data

All manuscripts must include a data availability statement. This statement should provide the following information, where applicable:

- Accession codes, unique identifiers, or web links for publicly available datasets
- A list of figures that have associated raw data
- A description of any restrictions on data availability

The MS/MS data have been deposited to the ProteomeXchange Consortium via jPOSTrepo (https://repository.jpostdb.org/) with the dataset identifier JPST000623 (PXD014527). The NGS data have been deposited to the NCBI SRA with BioProject ID PRJNA560477.

Field-specific reporting

Please select the one below that is the best fit for your research. If you are not sure, read the appropriate sections before making your selection.

Life sciences study design

All studies must disclose on these points even when the disclosure is negative.

Sample size	All experiments in vitro and in cell culture were performed in technical triplicates. For in vivo experiments, n= 3 or n= 5 mice were injected with NanoMEDIC particles for analysis. No sample size calculation was performed.
Data exclusions	No data were excluded for in this study, except for mouse time-course experiment in Fig. 6D, data after 160 days were excluded because the two out of five mice died due to senility.
Replication	Production of functional NanoMEDIC particles has been performed by more than 6 people in this study, and we have confirmed the editing efficiency in various human and mouse cells and loci in technical triplicate. We have also confirmed in vivo delivery of NanoMEDIC containing either luciferase protein or Sp-Cas9 RNP delivery in at least 3 mice.
Randomization	Randomization in this study was not performed.
Blinding	Blinding in this study was not performed.

Reporting for specific materials, systems and methods

We require information from authors about some types of materials, experimental systems and methods used in many studies. Here, indicate whether each material, system or method listed is relevant to your study. If you are not sure if a list item applies to your research, read the appropriate section before selecting a response.

Materials & experimental systems

n/a	Involved in the study
☐	☒ Antibodies
☐	☒ Eukaryotic cell lines
☒	☐ Palaeontology
☐	☒ Animals and other organisms
☒	☐ Human research participants
☒	☐ Clinical data

Methods

n/a	Involved in the study
☒	☐ ChIP-seq
☐	☒ Flow cytometry
☒	☐ MRI-based neuroimaging

Antibodies

Antibodies used	anti-VSV-G (Sigma, cat# V5507), anti-HIV p24 (Abcam, cat# ab9071), anti-FKBP12 (Clontech, cat# 635089), anti-FRB (Clontech, cat# 635091), anti-SpCas9 (Active Motif, cat#61577), anti-HA (Santa Cruz Biotechnologies, cat# sc-7392), anti-dystrophin (Abcam, cat# ab15277), anti-Myosin (Santa Cruz Biotechnologies, cat# sc-20641), HRP-linked secondary anti mouse IgG antibody (Cell Signaling Technology, cat# 7076), HRP-linked secondary anti rabbit IgG antibody (Cell Signaling Technology, cat# 7074).
Validation	Antibodies were all validated for western blotting according to data available on the manufacturers' homepage.

Eukaryotic cell lines

Policy information about cell lines

Cell line source(s)	404C2 and 1383D2 iPSCs were obtained from Dr. Keisuke Okita (Kyoto University) and Dr. Masato Nakagawa. U937 Cells were obtained from RIKEN Cell Bank. C2C12, FF13096NOR, FFDMD111 and FF12020 iPSCs were obtained from Dr. Hidetoshi Sakurai (Kyoto University). Jurkat Cells were obtained from Dr. Hirohide Saito (Kyoto University). Hu5 Cells were obtained from Dr. Naohiro Hashimoto (National Center for Geriatrics and Gerontology). HEK293T cells were obtained from Dr. James Ellis (University of Toronto). All the iPS cell lines used in this study were previously established.
Authentication	Cells were not authenticated.
Mycoplasma contamination	Mycoplasma testing was routinely performed every 2 months on cultured cells used in this study with a MycoALERT Mycoplasma Detection Kit (Lonza) to confirm negative for mycoplasma contamination.
Commonly misidentified lines (See ICLAC register)	No commonly misidentified cell lines were used in the study.

Animals and other organisms

Policy information about studies involving animals; ARRIVE guidelines recommended for reporting animal research

Laboratory animals	C57BL/6J (male, 7 weeks) mice were used for the luciferase protein delivery experiments by NanoMEDIC. CAG-Luc2-hDMD Ex45KI C57BL/6J (female, 6 weeks) mice were used to assess the exon skipping activity by the luciferase reporter gene. NOG-mdx (male, 4 weeks) were used to assess the exon skipping of endogenous mouse dystrophin gene. Mice were housed under SPF condition with free-water and free-food supply with 12 hour light cycle.
Wild animals	No wild animals were used in the study.
Field-collected samples	No field collected samples were used in the study.
Ethics oversight	All in vivo experiments were approved by the Institutional Animal Care and Use Committee in Takeda Pharmaceutical Company Limited (Approval number AU-00020951) and by the CiRA Animal Experiment Committee in Kyoto University (Approval number KEI19-125).

Note that full information on the approval of the study protocol must also be provided in the manuscript.

Flow Cytometry

Plots

Confirm that:

- [x] The axis labels state the marker and fluorochrome used (e.g. CD4-FITC).
- [x] The axis scales are clearly visible. Include numbers along axes only for bottom left plot of group (a 'group' is an analysis of identical markers).
- [x] All plots are contour plots with outliers or pseudocolor plots.
- [x] A numerical value for number of cells or percentage (with statistics) is provided.

Methodology

Sample preparation	Adherent human HEK293T cells, and mouse C2C12 cells were washed with PBS, and then removed from plates by 0.5% trypsin without phenol red. Cells were resuspended in PBS containing 5% FBS, and filtered through mesh cloth to remove larger cell clumps, into flow cytometry tubes. For suspension cells, U937 and Jurkat, cells were centrifuged to remove media with phenol red, washed with PBS containing 5% FBS 2 times, and then resuspended in PBS containing 5%FBS. The suspension cells were filtered through a mesh cloth to remove large cell clumps into flow cytometry tubes.
Instrument	BD LSRFortessa Flow Cytometer
Software	FlowJo
Cell population abundance	10,000 gated cells from a scatter plot comparing SSC and FSC were recorded.
Gating strategy	From the starting cell population, the cells were gated by SSC and FSC. From this population, the cells were analyzed for GFP expression against either SSC or against an unrelated color.

- [x] Tick this box to confirm that a figure exemplifying the gating strategy is provided in the Supplementary Information.
